# Factors affecting the achievement of fertility intentions in urban Nigeria: analysis of longitudinal data

**DOI:** 10.1186/s12889-017-4934-z

**Published:** 2017-12-11

**Authors:** Stella Babalola, Olamide Oyenubi, Ilene S. Speizer, Lisa Cobb, Akinsewa Akiode, Mojisola Odeku

**Affiliations:** 1 Center for Communication Programs/Department of Health Behavior and Society, Johns Hopkins Bloomberg School of Public Health, Baltimore, MD USA; 2 University of North Carolina, Chapel Hill, NC USA; 3 Nigerian Urban Reproductive Health Initiative, Abuja, Nigeria

**Keywords:** Fertility intentions, Determinants, Urban women, Nigeria

## Abstract

**Background:**

Maternal mortality ratio in Nigeria is among the highest in the world with an estimated 160 women dying every day of complications related to pregnancy and childbirth. In addition to appropriate management of complications related to pregnancy and childbirth, preventing unwanted pregnancies is an effective way to reduce maternal deaths. Identifying potentially modifiable factors associated with the achievement of fertility intentions is critical for developing behavior change interventions that will contribute to reducing maternal mortality.

**Methods:**

The data analyzed came from a longitudinal design with data collected in 2010/2011 and 2014 from the same group of women of reproductive age in six Nigerian cities. The data were collected as part of efforts to evaluate the effects of the Nigerian Urban Reproductive Health Initiative (NURHI). A total of 10,672 women were interviewed at the two points in time but the analyses in this manuscript were limited to 1921 in-union women who reported that they desired no more children at baseline in 2010/2011. The principal analytic method was multivariable regression adjusted for clustering at the enumeration area level. The analysis controlled for socio-demographic and household variables, ideational characteristics, and contraceptive use at baseline.

**Results:**

About two thirds of the women who desired no more children at baseline have neither had any children nor were pregnant at endline. The strongest predictors of the achievement of fertility intentions include parity, age, religion, residence, spousal communication about family size, perceived severity of another pregnancy, and spousal agreement about family size.

**Conclusion:**

A comprehensive strategy to help women avoid unwanted pregnancies should include efforts to increase women’s understanding about effective ways to prevent unplanned pregnancies and strengthen self-efficacy for contraceptive use. Promoting spousal communication about reproductive issues, engaging men, promoting smaller family sizes and changing pronatalist attitudes should also be part of such a strategy.

## Background

According to the 2013 Nigeria Demographic Health Survey, one in ten pregnancies in Nigeria were either mistimed or not wanted [33]. Yet, about 16% of married women who want to stop or postpone childbearing in Nigeria are currently not using any form of contraception [33]. This unmet need for contraception is even higher amongst unmarried, sexually active women (22%). In 2012, a study using data obtained from a nationally representative sample of 772 health facilities and interviews with 194 health care professionals demonstrated an even higher proportion of unintended pregnancies. The study estimated that there were 9.2 million births in Nigeria in 2012 with almost a quarter (24%) mistimed or unwanted. More than half (56%) of these unintended pregnancies were resolved by illegal induced abortion [13].

Globally, Nigeria accounts for 19% of all maternal deaths [56]. The maternal mortality ratio (MMR) in Nigeria in 2015 was estimated to be about 814 per thousand live births, the fourth highest in the world after Sierra Leone, Central African Republic and Chad. This 2015 MMR represents a decline of only about 2% per annum since 1990 and translates into 160 women dying every day from complications related to pregnancy or childbirth [56].

Most causes of maternal deaths can be prevented by improved management of pregnancy-related complications and the use of family planning [9, 23]. If all women who wanted to delay or stop childbirth used effective contraception to prevent unintended pregnancy, maternal death would decline by 67% and newborn death would decline by 77%. Family planning is a high impact, cost-effective health intervention that could save numerous lives, especially in developing countries [50].

Behavioral change theories and models including the Theory of Planned Behavior [7, 8], the theory of reasoned action [17, 18], the protection motivation theory [40] and the model of interpersonal behavior [54] all suggest that intentions are the most proximal and most important determinants of human behaviors. Intentions offer a more reliable predictor of human behavior compared to other cognitions including self-efficacy, attitudes, beliefs and norms and perception of risk severity [31, 44, 45]. Substantial evidence supports the association between fertility intentions and subsequent fertility ([12, 14, 29, 39, 42]; Ilene S. [52]). However, there is still a considerable gap between fertility intentions and actual behaviors to prevent a birth [3, 34]. This discrepancy is typically referred to as the “intention-behavior gap” where individual intentions do not necessarily translate to behavior.

The basis for forming an intention has been shown to predict how well the intention predicts behavior. Sheeran and colleagues suggest that attitudinally controlled intentions which are based on personal beliefs about the consequences of actions or self-chosen intentions predict actualization of the behaviors better than normatively controlled intentions which are based on social pressures to act [46]. However, Godin et al. argue that if a behavior has moral relevance, intentions that are based on moral norms are much stronger predictors of behavior than attitudinally controlled intentions [24]. When individuals experience a moral obligation to perform a particular behavior they are more tightly bound to their intentions, making it more likely for them to carry them out [1, 24, 25]. Individuals who anticipate significant regret if they do not carry out a behavior have a higher likelihood of carrying out the behavior than individuals with similar intentions who do not hold such regret [3].

Past behavior in itself has a bearing on the likelihood that a behavior would be performed [35]. However, other research has shown that past behavior moderates the relationship between intentions and behaviors. Kashima and colleagues showed that intentions that are based on greater experience predict behaviors better than those with lesser experience [30]. Also, Sheeran, Orbell and Tramifow have shown that intentions are more stable when they are formed from behaviors for which individuals have greater experience and therefore produce a stronger intention-behavior correlation [47].

Some studies have emphasized the importance of implementation intentions for behaviors. According to the studies in this school of thought, promoting implementation intentions that emphasize specific actions required for goal implementation can more easily bridge the intention-behavior gap. Thus, planning upfront what, when, and how to take actions favors the attainment of the goal [26–28, 48, 55]. Other studies have argued that level of goal commitment and strength of intentions are critical elements in translating intentions to behaviors [19, 38, 43].

Literature abounds with evidence about the factors associated with the achievement of reproductive intentions. These studies have identified marital duration, age at first marriage, family size and education to be strong determinants of achievement of fertility intentions [4, 5, 16, 21]. Other documented predictors of reproductive intentions achievement include spousal communication, the level of agreement between the husband and the wife regarding family size, husband’s reproductive intentions, and recent loss of a child [21, 22]. Furthermore, some studies have found that the woman’s age, autonomy and religion are significant predictors [4–6]. Others have stressed the negative role of intimate partner violence [20, 37, 49].

Understanding the potentially modifiable factors associated with acting on fertility intentions in the Nigerian context is key to creating interventions than can facilitate the uptake of healthy behaviors and lead to reduced unintended pregnancies. Using a prospective longitudinal sample of Nigerian women matched at baseline and endline, this study examines the factors that are associated with the achievement of intentions amongst women who reported that they wanted to stop childbearing in six urban sites of Nigeria.

The context for this research was an impact evaluation of a comprehensive family planning program, the Nigerian Urban Reproductive Health Initiative (NURHI), that aimed to substantially increase the contraceptive prevalence rate. The project did this by addressing supply and demand barriers to contraceptive use in six urban centers of Nigeria: Abuja, Benin, Kaduna, Ibadan, Ilorin, and Zaria. NURHI was implemented between 2009 and 2015 by the Johns Hopkins Center for Communication Programs and its Nigerian partners with funding from the Bill & Melinda Gates Foundation.

## Methods

### Participants

The data analyzed in this manuscript come from a longitudinal sample of women interviewed in 2010 (baseline) and again in 2014 (endline). The data were collected by the Measurement, Learning & Evaluation project as part of efforts to evaluate NURHI. Survey respondents were first selected in 2010 following a two-stage sampling design that involved first selecting primary sampling units (PSU) with probability proportional to size and subsequently households within each PSU. A more detailed description of the sampling procedure has been provided elsewhere [32]. The original dataset includes 10,672 women matched with information at baseline (2010/2011) and endline (2014). The analyses presented here focused on 1921 women who were in-union at baseline and who reported at baseline an intention not to have any more children. Women who reported that they were unable to get pregnant were excluded from the analysis.

### Variables

#### Dependent variable

The dependent variable is achievement of reproductive intention, defined as no birth or pregnancy in the nearly four-year follow-up period between baseline and endline.

#### Independent variables

Consistent with extant literature on the predictors of unintended pregnancy and achievement of reproductive intentions, we assessed the predictive value of the following independent variables:

I. Age at baseline: Defined as age in single years at baseline. Only women aged between 15 and 49 years were eligible for inclusion in the surveys.
II. Parity at baseline: Defined as the number of children the woman ever had at baseline. Parity varied between 0 and 15 at baseline.
III. Religion: We distinguished between Christians and Muslims.
IV. Education level at baseline: Defined as formal education. Women were classified into one of four categories: no formal education, primary, secondary, and higher.
V. Spousal restrictions on wife’s movement and relationships: This construct was derived from six questions asked of women on whether the woman’s husband prohibited her from: working outside the home, receiving visits from people, visiting friends, visiting family, using contraceptives, or using mobile phones. The six items had a Cronbach’s alpha score of 0.787. Overall, 27.3% of the respondents reported at least one spousal prohibition.
VI. Level of spousal agreement on the number of children to have: This was assessed as a categorical variable based on women’s reports that classified the women into four groups: the woman reported that she wanted same number of children as her husband, the woman reported that she wanted fewer children than her husband, the women reported that she wanted more children than her husband, and the woman did not know how many children her husband wanted.
VII. Prior discussion of family size with spouse at baseline: Defined as ever discussing the desired number of children with spouse at baseline.
VIII. Perception at baseline that having another child would be a big problem: Derived from a baseline question that asked the respondents how big of a problem discovering that they were pregnant in the next few weeks would be. Respondents had the response options of “a big problem”, “a small problem” or “no problem at all”. In the analysis, we distinguished between women that perceived a pregnancy to be a big problem and others.
IX. Use of modern method at baseline: We distinguished between women who were using a modern method (including female sterilization, vasectomy, implants, IUD, injectables, daily pill, diaphragm, emergency pill, male condom, female condom, and lactational amenorrhea method) at baseline and those who were using either a traditional method or no method at all.
X. Household wealth: An asset-based construct divided into quintiles. Following Rutstein and Johnson [41] we computed wealth quintiles though principal component analysis of selected household assets and housing characteristics.
XI. City of residence: Defined as the place of residence at baseline.

### Analysis

We used two analytic methods in this manuscript: unadjusted and adjusted logistic regression. The unadjusted logistic regression examined the bivariate relationship between the dependent variable and each of the independent variables. The adjusted logistic regression controlled for all the independent variables listed above. The regression models were adjusted for potential violation of independence assumption at the cluster level. The analyses were performed in Stata 14 [53].

## Results

The socio-demographic, ideational and behavioral characteristics of the women who wanted no more children are presented in Table 1. About two-thirds (65.1%) of the women that did not want any more children at baseline achieved their fertility intentions. At endline in 2014, these women had not had an additional birth and were not currently pregnant. The average age at baseline was 38.8 years while the mean parity was 5.3 children. The majority of the respondents had some form of formal education and the modal education level was secondary. Spousal communication about family size was reported by 61.9% of the respondents at baseline. Only about a third (33.2%) of the respondents were using a modern method of contraception at baseline, mainly injectables (38.1%), intrauterine device (20.2%), condom (16.3%), and daily pill (14.3%). The respondents were approximately equally divided between Muslims and Christians. Similarly, the respondents were equally divided among those who perceived that another pregnancy would be a big problem and those that did not. Proportionally more of the women who did not want any more children at baseline were from Ibadan and Ilorin than any other city. The data further show that the respondents were more likely to be in the higher wealth groups than expected, indicating that poor women were more likely than richer ones to desire additional children. In additional, about three fifths of the study participants reported desiring as many children as their spouse. Finally, about three-quarters of the women reported no spouse-imposed restrictions.

**Table 1:** Socio-demographic, ideational and behavioral characteristics of study participants (women who desired no additional children at baseline)

Respondents’ Characteristics	*N*	Unweighted %/Mean	Weighted %/Mean
Achievement of fertility desires of no additional child			
Achieved – no additional child or pregnancy	1254	65.3	65.1
Did not achieve – had an additional child or pregnancy	667	34.7	34.9
Discussion of family size with spouse/partner at baseline			
Discussed	1167	60.8	61.9
Did not discuss	754	39.2	38.1
Using modern method at baseline			
Yes	645	33.6	33.2
No	1276	66.4	66.8
City of residence at baseline			
Abuja	252	13.1	13.6
Benin City	241	12.6	11.9
Ibadan	423	22.0	21.7
Ilorin	446	23.2	22.7
Kaduna	269	14.0	14.8
Zaria	290	15.1	15.3
Education Level at baseline			
No formal education	365	19.0	17.9
Primary	447	23.3	23.5
Secondary	660	34.3	36.0
Higher	449	23.4	22.6
Religion at baseline			
Christian	949	49.4	51.3
Muslim	972	50.6	48.7
Wealth Quintile at baseline			
Lowest	277	14.4	14.5
Second	344	17.9	17.8
Middle	397	20.7	21.3
Fourth	407	21.2	21.9
Highest	496	25.8	24.5
Perception that a pregnancy would be a big problem at baseline			
Perceived	955	49.7	51.2
Did not perceive	966	50.3	48.8
Spousal agreement about number of children to have as reported by the woman at baseline			
Husband wants same number as wife	1166	61.2	60.9
Husband wants more children than wife	301	15.8	15.7
Husband wants fewer children than wife	114	6.0	6.6
Does not know how many children husband wants	325	17.0	16.8
Husband’s restrictions on wife’s movement and relationships as reported by woman at baseline			
At least one restriction	524	27.3	26.4
No restriction	1397	72.7	73.6
Mean (SD) number of children ever born at baseline	1921	5.4 (2.4)	5.3 (2.3)
Mean (SD) age in years at baseline	1921	39.3 (5.8)	38.8 (5.9)

The unadjusted logistic regression results (Table 2) show that the socio-demographic variables most strongly associated with achievement of fertility intentions were parity, age, religion, city of residence, education, and wealth quintile. The significant behavioral and ideational correlates revealed by the unadjusted results were spousal communication about family size, the perception that another pregnancy would be a big problem, and baseline use of a modern method at baseline.

**Table 2:** Results of the logistic regression of achievement of fertility intention of no additional child on ideational and socio-demographic variables

Independent Variables	Unadjusted Odds Ratio (95% CI)	Adjusted Odds Ratio (95% CI)	Fully Standardized Beta
Ever discussed family size with spouse (RC = Never discussed)	1.488*** (1.229, 1.800)	1.487** (1.137, 1.944)	0.097
Using any modern method at baseline (RC = Not using)	1.654*** (1.354, 2.021)	1.467*** (1.167, 1.843)	0.091
Number of children-ever-born at baseline	1.143*** (1.091, 1.197)	1.294*** (1.208, 1.389)	0.307
Age at baseline	1.088*** (1.069, 1.106)	1.066*** (1.047, 1.086)	0.187
Believed at baseline that having another child would be a big problem (RC = Did not so believe)	1.440*** (1.185, 1.750)	1.319** (1.070, 1.627)	0.069
Muslim (RC = Christian)	0.693*** (0.574, 0.836)	0.715** (0.562, 0.909)	−0.084
Spousal agreement about number of children to have as reported by the woman at baseline			
Husband desires same number as wife (RC)	1.00	1.00	–
Husband desire more children than wife	0.678**(0.522, 0.880)	0.806 (0.579, 1.120)	−0.039
Husband desired fewer children than wife	0.876 (0.577, 1.330)	0.800 (0.513, 1.247)	−0.026
Does not know how many children husband desires	0.625*** (0.482, 0.810)	0.806 (0.578, 1.124)	−0.041
Husband placed any restrictions on wife’s movement and relationships; reported by woman at baseline (RC = No restrictions)	0.797* (0.639, 0.995)	0.966 (0.750, 1.243)	−0.008
City of Residence			
Abuja (RC)	1.00	1.00	–
Benin City	0.665* (0.431, 1.025)	0.618* (0.393, 0.973)	−0.079
Ibadan	0.760 (0.517, 1.115)	0.879 (0.589, 1.312)	−0.027
Ilorin	0.655* (0.448, 0.957)	0.848 (0.568, 1.267)	−0.035
Kaduna	0.729 (0.486, 1.093)	0.738 (0.484, 1.124)	−0.053
Zaria	0.606** (0.412, 0.891)	0.515** (0.308, 0.859)	−0.119
Education Level at Baseline			
No formal education (RC)	1.00	1.00	–
Primary	0.886 (0.653, 1.203)	0.925 (0.648, 1.322)	−0.016
Secondary	1.046 (0.778, 1.407)	1.173 (0.800, 1.721)	0.038
Higher	1.681*** (1.235, 2.289)	1.610* (1.042, 2.489)	0.101
Baseline Wealth Quintile			
Lowest (RC)	1.00	1.00	–
Second	1.010 (0.730, 1.397)	0.932 (0.665, 1.305)	−0.014
Middle	1.136 (0.831, 1.552)	1.061 (0.757, 1.487)	0.012
Fourth	1.322‡ (0.962, 1.817)	1.106 (0.785, 1.558)	0.021
Highest	1.778*** (1.293, 2.444)	1.417‡ (0.971, 2.066)	0.077
Pseudo-R^2^	–	10.1%	–
Goodness of fit ***X*** ^2^/p	–	1904.2/0.253	–

*RC* Reference Category

‡ *p* ≤ 0.1; * ≤ 0.05; ** ≤ 0.01; *** ≤ 0.001

The results of the multivariable logistic regression (Table 2) confirmed the bivariate findings although the effects of some variables are attenuated. The odds of achieving fertility intentions were about 50% higher for women who had ever discussed family size with their spouses compared to their peers that had not. Similarly, use of modern contraceptive methods at baseline increased the odds of achieving fertility intentions by 46.7%. The differences by city of residence were such that the respondents from Benin City and Zaria were less likely than their peers from Abuja to achieve their reproductive intentions. Muslims were 29% less likely than Christians to attain their fertility desires. Women who perceived another pregnancy to be a big problem were about 32% more likely than those who did not perceive this occurrence to be a big problem to achieve their fertility intentions. The relationship with parity was positive such that a unit increase in the number of children ever born was associated with 29% increase in the odds of achieving fertility intentions. Similarly, the relationship with age was positive with a unit increase in age associated with an increase of 6.6% in the odds of achieving fertility intentions. The difference by level of education was only significant when we compare no formal education with tertiary education: the odds of achieving fertility intentions were 61% higher for women with tertiary education than for women with no formal education. Similarly, the difference by wealth quintile was only significant (and only marginally) if we compare the lowest and the highest quintile: the women in the highest quintile were about 42% more likely to achieve their fertility intentions compared to the women in the lowest quintile. Finally, spousal agreement about the number of children and spousal restrictions made no significant difference.

A look at the fully standardized beta weights reveals that the most important correlates of achieving fertility intentions are parity, woman’s age, spousal communication about family size, city of residence, and higher education.

## Discussion

This manuscript examined the factors associated with the achievement of fertility intentions to stop childbearing. The analyses were based on longitudinal data, which helps to correct for major threats to internal validity and strengthens causal claims. It is interesting to note that the women who desired no more children were, on average, near the end of their childbearing age and were of higher parity. In other words, the data showed that urban women in Nigeria do not desire to stop childbearing until they have reached an advanced age and have a relatively large number of children. These findings are reflective of a high fertility setting where pronatalist attitudes are prevalent and are reminiscent of what other studies in Nigeria have suggested [6, 36]. These two variables – age and parity – were also the most important correlates of achievement of fertility intentions among these women who desired no more children. In other words, the greatest motivations for not having another child or pregnancy were the age of the woman and the number of children that she currently had. This finding is consistent with results from other studies [4, 5, 16]. Furthermore, the finding underscores the importance of promoting smaller family sizes and changing pronatalist attitudes.

Another strong and positive predictor of achievement of fertility desires was discussion of family size with spouse. Specifically, the women who discussed the number of children with their spouse or partner were more likely than the other women to achieve their fertility intention. The implication of this finding is that promoting spousal communication about reproductive issues and engaging men should be part of a comprehensive strategy to prevent unplanned pregnancies. For example, communication materials that show a man and his wife discussing family planning and deciding to adopt a method are relevant.

Consistent with the literature on the role of attitudinally-based intentions on behaviors [1, 2, 15, 24, 46, 47], the perception that another pregnancy would be a big problem significantly and positively predicted the achievement of fertility intentions. It makes intuitive sense that women who perceived severe consequences of another pregnancy would be more determined than others to avoid a pregnancy. Related to this finding is the result that women who were using a modern method at baseline were more likely than others to achieve their reproductive intentions. Using an effective method of contraception reflects an appreciable level of determination to avoid a pregnancy [52]. This finding echoes results of studies that have linked goal commitment and the strength of intentions with goal achievement [19, 38, 43]. The programmatic implications of the documented roles of perceived severity of the consequences of another pregnancy and prior contraceptive use include the need to increase women’s understanding of effective ways to prevent unwanted pregnancies and strengthen self-efficacy for contraceptive use. Literature abounds with evidence on strategies for strengthening self-efficacy for action [11]. Relevant strategies include identifying and removing logistic and structural barriers to contraceptive access; promoting discussion about contraceptive use; coaching men and women to communicate with their spouses about contraception using a satisfied contraceptive user similar to the intended audience to model relevant behaviors; addressing fears about contraceptives; and correcting misconceptions [10].

These findings come in the context of an evaluation of a program that was designed specifically to positively change modifiable factors that are predictive of contraceptive use. Most relevant to this discussion was a focus on increasing spousal communication on family size and contraception. The program also focused on changing ideational factors such as contraceptive knowledge and self-efficacy to use contraceptives despite social disapproval, as well as improving service availability and quality. Program impact was significant, with measurable change in contraceptive prevalence rate, intention to use contraceptives, and ideational factors predictive of contraceptive use associated with program exposure [32].

This study has a few limitations that warrant mention. Whereas the use of longitudinal data helps to strengthen causal claims, the data analyzed in this manuscript are derived from self-reported information and are likely affected by social desirability bias and memory lapse. During data collection, fieldworkers took appropriate steps to minimize social desirability bias, including private one-on-one interviews without the presence of a third party, assurance of confidentiality, voluntary participation, and informed consent. Second, this study only includes those women who at baseline reported that they do not want any more children. Prior research has demonstrated that fertility desires are fluid and not static [51]. Therefore some women in the sample may have changed their fertility desires over the follow-up period and decided that they wanted more children. In addition, other women not included in the sample may have, after the baseline, decided that they did not want any more children but are not included here. In this analysis, we do not account for these changing fertility desires. Furthermore, the modifiable predictor variables included in the analysis are also subject to change over time. Since these variables were measured at baseline, it is possible that some of them may have changed before the final assessment. The analysis did not account for these changes and their potential effects on fertility desires.

Despite these limitations, this study that uses longitudinal data provides insights into approaches that can be used in urban Nigeria to support women to attain their desire to stop childbearing and reduce unintended pregnancies. Programs that promote couple communication, male involvement, and support attainment of fertility desires through the mass media or community-based outreach activities will help women (and couples) to achieve their fertility desires and help Nigeria to reduce the currently high maternal mortality ratio.

## Conclusion

Using panel data, this study has identified a number of potentially modifiable factors associated with the achievement of reproductive goals among Nigerian urban women. A comprehensive strategy to help women avoid unwanted pregnancies should include efforts to increase women’s understanding about effective ways to prevent unplanned pregnancies and strengthen the self-efficacy for contraceptive use. Promoting spousal communication about reproductive issues, engaging men, promoting smaller family sizes and changing pronatalist attitudes should also be part of such a strategy.

## References

[1] Abraham C, Sheeran P (2003). Acting on intentions: the role of anticipated regret. Br J Soc Psychol.

[2] Abraham C, Sheeran P (2004). Deciding to exercise: the role of anticipated regret. Br J Health Psychol.

[3] Abraham C, Sheeran P, Norman P, Conner M, Vries N (1999). d, Otten W. When good intentions are not enough: modeling Postdecisional cognitive correlates of condom Use. J Appl Soc Psychol.

[4] Adebowale SA, Adeoye IA, Palamuleni ME (2013). Contraceptive use among Nigerian women with no fertility intention: interaction amid potential causative factors. Etude de la Population Africaine.

[5] Adhikari R, Soonthorndhada K, Prasartkul P (2009). Correlates of unintended pregnancy among currently pregnant married women in Nepal. BMC Int Health Hum Rights.

[6] Adiri F, Ibrahim HI, Ajayi V, Sulayman HU, Yafeh AM, Ejembi CL (2010). Fertility behaviour of men and women in three communities in Kaduna state, Nigeria. Afr J Reprod Health.

[7] Ajzen I, Kuhl J, Beckmann J (1985). From intentions to actions: a theory of planned behavior. Action control: from cognition to behavior.

[8] Ajzen I (1991). The theory of planned behavior. Organ Behav Hum Decis Process.

[9] Alkema L, Chou D, Hogan D, Zhang S, Moller A-B, Gemmill A (2016). Global, regional, and national levels and trends in maternal mortality between 1990 and 2015, with scenario-based projections to 2030: a systematic analysis by the UN maternal mortality estimation inter-agency group. Lancet.

[10] Babalola S, John N, Ajao B, Speizer IS (2015). Ideation and intention to use contraceptives in Kenya and Nigeria. Demogr Res.

[11] Bandura A (1977). Self-efficacy: toward a unifying theory of behavioral change. Psychol Rev.

[12] Bankole A (1995). Desired fertility and fertility behaviour among the Yoruba of Nigeria: a study of couple preferences and subsequent fertility. Popul Stud.

[13] Bankole A, Adewole IF, Hussain R, Awolude O, Singh S, Akinyemi JO (2015). The incidence of abortion in Nigeria. Int Perspect Sex Reprod Health.

[14] Bankole A, Westoff CF (1998). The consistency and validity of reproductive attitudes: evidence from Morocco. J Biosoc Sci.

[15] Conner M, Sandberg T, McMillan B, Higgins A (2006). Role of anticipated regret, intentions and intention stability in adolescent smoking initiation. Br J Health Psychol.

[16] De Silva WI (1992). Achievement of reproductive intentions in Sri Lanka, 1982–1985: a longitudinal study. Soc Biol.

[17] Fishbein M. A theory of reasoned action: some applications and implications. In: Herbert E. Howe & M. M. Page (Eds.), Nebraska Symposium on Motivation, 1979, Volume 27: Attitudes, Values, and Beliefs (Vol. 27, pp. 65-116). Lincoln: University of Nebraska Press; 1980. 7242751

[18] Fishbein M, Ajzen I (1975). Belief, attitude, intention and behavior: an introduction to theory and research.

[19] Fuchs R, Seelig H, Göhner W, Schlatterer M, Ntoumanis N (2017). The two sides of goal intentions: intention self-concordance and intention strength as predictors of physical activity. Psychol Health.

[20] Gao W, Paterson J, Carter S, Iusitini L (2008). Intimate partner violence and unplanned pregnancy in the Pacific Islands families study. Int J Gynecol Obstet.

[21] Geda NR, Lako TK (2012). Unintended pregnancy among married women in Damot Gale District, southern Ethiopia: examining the prevalence and risk factors. Afr Popul Stud.

[22] Gipson JD, Hindin MJ (2009). The effect of husbands’ and wives’ fertility preferences on the likelihood of a subsequent pregnancy, Bangladesh 1998–2003. Popul Stud.

[23] Glasier A, Gülmezoglu AM, Schmid GP, Moreno CG, Van Look PF (2006). Sexual and reproductive health: a matter of life and death. Lancet.

[24] Godin G, Conner M, Sheeran P (2005). Bridging the intention–behaviour gap: the role of moral norm. Br J Soc Psychol.

[25] Godin G, Germain M, Conner M, Delage G, Sheeran P (2014). Promoting the return of lapsed blood donors: a seven-arm randomized controlled trial of the question–behavior effect. Health Psychol.

[26] Gollwitzer PM (1999). Implementation intentions: strong effects of simple plans. Am Psychol.

[27] Gollwitzer PM, Sheeran P (2006). Implementation intentions and goal achievement: a meta-analysis of effects and processes. Adv Exp Soc Psychol.

[28] Hagger MS, Luszczynska A (2014). Implementation intention and action planning interventions in health contexts: state of the research and proposals for the way forward. Appl Psychol Health Well Being.

[29] Islam MM, Bairagi R (2003). Fertility intentions and subsequent fertility behaviour in Matlab: do fertility intentions matter?. J Biosoc Sci.

[30] Kashima Y, Gallois C, McCamish M (1993). The theory of reasoned action and cooperative behaviour: it takes two to use a condom. Br J Soc Psychol.

[31] McEachan RRC, Conner M, Taylor NJ, Lawton RJ (2011). Prospective prediction of health-related behaviours with the theory of planned behaviour: a meta-analysis. Health Psychol Rev.

[32] Measurement Learning and Evaluation Project Nigeria Team (2017). Evaluation of the Nigerian urban reproductive health initiative (NURHI) program. Stud Fam Plan.

[33] National Population Commission (NPC) [Nigeria] and ICF International (2014). Nigeria demographic and health survey 2013.

[34] Orbell S, Sheeran P (1998). ‘Inclined abstainers’: a problem for predicting health-related behaviour. Br J Soc Psychol.

[35] Ouellette JA, Wood W (1998). Habit and intention in everyday life: the multiple processes by which past behavior predicts future behavior. Psychol Bull.

[36] Oyediran KA (2006). Fertility desires of Yoruba couples of south-western Nigeria. J Biosoc Sci.

[37] Pallitto CC, García-Moreno C, Jansen HA, Heise L, Ellsberg M, Watts C (2013). Intimate partner violence, abortion, and unintended pregnancy: results from the WHO multi-country study on Women’s health and domestic violence. Int J Gynecol Obstet.

[38] Prestwich A, Kellar I (2014). How can the impact of implementation intentions as a behaviour change intervention be improved?. Revue Européenne de Psychologie Appliquée/European Review of Applied Psychology.

[39] Razzaque A (2000). Preference for children and subsequent birth: evidence from Matlab, Bangladesh. Genus.

[40] Rogers RW, Cacioppo JT, Petty RE (1983). Cognitive and psychological processes in fear appeals and attitude change: A revised theory of protection motivation. Social psychophysiology: A sourcebook.

[41] Rutstein SO, Johnson K (2004). The DHS wealth index. DHS comparative reports no. 6.

[42] Schoen R, Astone NM, Kim YJ, Nathanson CA, Fields JM (1999). Do fertility intentions affect fertility behavior?. J Marriage Fam.

[43] Seo E, Patall EA, Henderson MD, Steingut RR. The Effects of Goal Origin and Implementation Intentions on Goal Commitment, Effort, and Performance. The Journal of Experimental Education. doi:10.1080/00220973.2016.1277334.

[44] Sheeran P, Harris PR, Epton T (2014). Does heightening risk appraisals change people’s intentions and behavior? A meta-analysis of experimental studies. Psychol Bull.

[45] Sheeran P, Klein WM, Rothman AJ (2016). Health behavior change: moving from observation to intervention. Annu Rev Psychol.

[46] Sheeran P, Norman P, Orbell S (1999). Evidence that intentions based on attitudes better predict behaviour than intentions based on subjective norms. Eur J Soc Psychol.

[47] Sheeran P, Orbell S, Trafimow D (1999). Does the temporal stability of behavioral intentions moderate intention-behavior and past behavior-future behavior relations?. Personal Soc Psychol Bull.

[48] Sheeran P, Webb TL, Gollwitzer PM (2005). The interplay between goal intentions and implementation intentions. Personal Soc Psychol Bull.

[49] Silverman JG, Gupta J, Decker MR, Kapur N, Raj A (2007). Intimate partner violence and unwanted pregnancy, miscarriage, induced abortion, and stillbirth among a national sample of Bangladeshi women. BJOG.

[50] Singh S, Darroch JE, Ashford LS (2014). Adding it up: the costs and benefits of investing in sexual and reproductive health 2014.

[51] Speizer IS, Calhoun LM, Hoke T, Sengupta R (2013). Measurement of unmet need for family planning: longitudinal analysis of the impact of fertility desires on subsequent childbearing behaviors among urban women from Uttar Pradesh, India. Contraception.

[52] Speizer IS, Lance P (2015). Fertility desires, family planning use and pregnancy experience: longitudinal examination of urban areas in three African countries. BMC Pregnancy Childbirth.

[53] Statacorp (2015). Stata Base reference manual release 14.

[54] Triandis HC. Values, attitudes, and interpersonal behavior. In: Herbert E. Howe & M. M. Page (Eds.), Nebraska symposium on motivation, 1979, Volume 27: Attitudes, Values, and Beliefs (pp. 195-259). Lincoln: University of Nebraska Press; 1980. 7242748

[55] Webb TL, Sheeran P (2007). How do implementation intentions promote goal attainment? A test of component processes. J Exp Soc Psychol.

[56] World Health Organization, UNICEF, UNFPA, & The World Bank (2016). Trends in maternal mortality: 1990 to 2015: estimates by WHO, UNICEF, UNFPA, the World Bank and the United Nations population division.

